# Anti-synthetase syndrome with anti-PL-7 antibody positive in a child: a case report and literature review

**DOI:** 10.3389/fimmu.2025.1525432

**Published:** 2025-03-03

**Authors:** Jia Liu, Nana Nie, Ranran Zhang, Dahai Wang, Yi Lin, Hong Chang

**Affiliations:** Deparment of Pediatric Nephrology, Rheumatology and Immunity, The Affiliated Hospital Of Qingdao University, Qingdao, China

**Keywords:** anti-synthetase syndrome, anti-PL-7 antibody, myositis, case report, ILD

## Abstract

**Background:**

Anti-synthetase syndrome (ASS) is a rare autoimmune myopathy and forms part of the idiopathic inflammatory myopathies (IIMs). A distinctive feature of ASS is the presence of anti-aminoacyl tRNA synthase (ARS) antibodies, which target synthetases, leading to inflammation in muscles, lungs, and other tissues.

**Case presentation:**

A case of a 12-year-old Chinese girl with recurrent fever, myalgia, and Gottron’s papules is reported. Serum creatine kinase was markedly elevated, and chest CT revealed interstitial changes. Magnetic Resonance Imaging (MRI) of the left thigh indicated soft tissue swelling and interstitial fluid accumulation. Electromyography demonstrated myogenic injury. Pathological examination of a left thigh muscle biopsy revealed local edema, focal lymphocyte infiltration, and proliferation and dilation of interstitial small vessels. The myositis antibody spectrum test was positive for anti-PL-7 antibodies. Treatment with glucocorticoids and methotrexate led to significant improvement in her condition.

**Conclusion:**

This case represents the youngest reported patient with PL-7 positive ASS to date.

## Background

Anti-synthetase syndrome (ASS) is a rare autoimmune myopathy classified under idiopathic inflammatory myopathies (IIMs). A distinctive feature of ASS is the presence of anti-aminoacyl tRNA synthase (ARS) antibodies, which target synthetases, causing inflammation in muscles, lungs, and other tissues (1). This syndrome involves multiple systems and commonly presents with muscle weakness, interstitial lung disease (the most prevalent complication), arthritis, Raynaud’s phenomenon, ‘mechanic’s hands,’ and fever. The prognosis of ASS varies, with early diagnosis and treatment being crucial for a favorable outcome. The presence and severity of interstitial lung disease are critical to the prognosis (2, 3). ASS predominantly affects adults, usually diagnosed between 40-60 years, with an estimated incidence of 1 to 1.5 cases per million annually. Pediatric cases are significantly rarer (4).

Here, a case of an ASS child is reported, who presented with recurrent fever, myositis, and erythematous patches on the extensor surfaces of the elbows. Myositis antibody testing indicated positive anti PL-7 antibodies. This patient is the youngest reported case with PL-7 positive ASS to date.

## Case presentation

**Figure 1:**
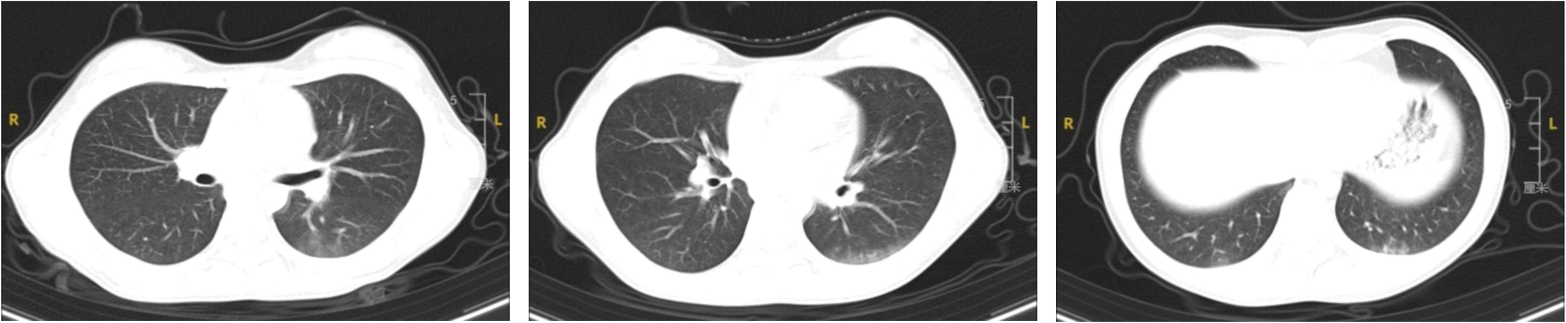
Chest CT findings of the patient.

A 12-year-old girl was admitted to the hospital on April 7, 2024, presenting with recurrent fever of unknown origin lasting nine days. Prior to admission, her fever had reached a peak of 38.9°C daily without additional symptoms. Initial blood tests at another hospital indicated a white blood cell count of 30.1 × 10^9^/L, a neutrophil count of 23.52 × 10^9^/L, and a CRP level of 13.66 mg/L. Both hemoglobin concentration and platelet count were within normal limits, and tests for Mycoplasma pneumoniae antibodies were negative. Chest X-ray findings were normal. Following treatment with intravenous cefoperazone sodium and sulbactam sodium, her temperature decreased to 37.5°C. Subsequent tests showed fluctuations in white blood cell count (17.83-24.5 × 10^9^/L), neutrophil count (13.53-19.41 × 10^9^/L), and CRP levels (16.50-22.03 mg/L). She continued to experience recurrent fevers and occasional coughs. Her medical, personal, and family histories were unremarkable. Physical examination upon admission did not reveal any significant findings. Further laboratory tests showed marked leukocytosis (15-30 × 10^9^/L), predominantly neutrophils, mild anemia (106-126 g/L), elevated erythrocyte sedimentation rate (49 mm/h), and increased levels of CRP (13-28 mg/L), procalcitonin, and ferritin. Creatine kinase was raised to 762 U/L, with alanine aminotransferase levels between 47-69 U/L, and aspartate aminotransferase levels between 36-51 U/L. Direct Coombs test and antinuclear antibody (ANA) test were positive, with an ANA titer of 1:320. Anti-Ro52 antibodies were suspected to be positive on the Extractable Nuclear Antigen (ENA) enzyme spectrum, however, other immune indicators and pathogen tests returned negative results. A chest Computed Tomography (CT) revealed multiple ground-glass opacities beneath the pleura of both lungs, with indistinct borders (**Figure 1**). No abnormalities were detected in bone marrow aspiration, ECT, echocardiography, or major vessel ultrasound. On the ninth day of her hospital stay, she developed pain in her left leg muscle. MRI of the left thigh displayed soft tissue swelling and interstitial fluid accumulation. Electromyography suggested myogenic damage, and the myositis antibody test (immunoblotting) was positive for the PL-7 antibody. Pathological examination of a biopsy from the left thigh muscle showed localized edema, focal lymphocyte infiltration, and interstitial small vessel proliferation and dilation. On May 29, 2024, she developed a light purple rash on the extensor sides of both elbows. This patient has been diagnosed with anti-synthetase syndrome with anti-PL-7 antibody positive. Treatment included oral prednisone acetate (20 mg twice daily, approximately 1 mg/kg), methotrexate (12.5 mg weekly, approximately 10 mg/m^2^), folic acid tablets, and calcium. Following treatment, her body temperature normalized, and the thigh muscle pain resolved. The dosage of corticosteroids is currently being tapered.

## Discussion

IIMs are characterized by non-purulent muscle inflammation and muscle weakness (5). Subtypes of IIM include polymyositis (PM), dermatomyositis (DM), and inclusion body myositis (IBM), predominantly observed in adults, whereas juvenile dermatomyositis (JDM) is more prevalent among children. ASS is recognized as a subset of PM/DM (6).

Notably, unlike adult-onset IIM, most pediatric cases, particularly JDM, lack myositis-specific autoantibodies, which is a key distinguishing feature between childhood and adult IIMs. Studies indicate that up to 50% of JDM patients do not express known myositis-specific antibodies, in contrast to adult DM patients, where the seropositivity rate is significantly higher (7). This discrepancy underscores the importance of comprehensive clinical evaluation in pediatric cases where serological markers may be absent.

The diagnostic criteria for IIMs, established by Bohan and Peter, are widely employed in clinical settings (8, 9). In 2017, the European League Against Rheumatism (EULAR) and the American College of Rheumatology (ACR) introduced more stringent and clinically pertinent revised classification and diagnostic criteria for IIMs in adults and adolescents (6). These criteria integrate clinical, histopathological, and serological findings, enhancing diagnostic accuracy. However, given the lower prevalence of myositis-specific autoantibodies in children, their application in pediatric populations, particularly for JDM, may require further validation and adaptation.

ASS was initially described by Marguerie et al. in 1990 (10), and its hallmark clinical features are associated with the presence of anti-tRNA synthetase antibodies. Patients with ASS often exhibit a spectrum of clinical and laboratory signs, however, a unified diagnostic standard for ASS has not been established in clinical practice. In 2010, Connor et al. first formally suggested potential diagnostic criteria for ASS (11), followed by more rigorous criteria proposed by Solomon et al. in 2011. The criteria for diagnosis (12) include the presence of an anti-aminoacyl-tRNA synthetase antibody plus two major criteria or one major and two minor criteria. Major criteria include: (1) interstitial lung disease (ILD) not explained by environmental, occupational, or drug exposures and unrelated to any other underlying disease, and (2) polymyositis or dermatomyositis meeting the Bohan & Peter criteria. Minor criteria include: (1) arthritis, (2) Raynaud’s phenomenon, (3) mechanic’s hands. While arthritis, mechanic’s hands, and Raynaud’s phenomenon support the diagnosis, they are not essential for it.

Currently, 20 anti-ARS antibodies have been identified, with 8 being closely associated with ASS, including anti-Jo-1 (histidyl-tRNA synthetase), the first to be discovered and characterized (13); anti-PL-7 (threonyl); anti-PL-12 (alanyl); anti-EJ (glycyl); anti-OJ (isoleucyl); anti-KS (asparaginyl); anti-Zo (phenylalanyl); and anti-Ha (tyrosyl) (14, 15). Anti-PL-7 is among the rarest.

Studies in Japan and France have demonstrated that all patients with anti-PL-7 positive ASS exhibit concurrent ILD, with myositis occurring in approximately 50% to 86% of cases (16–18). Labirua-Iturburu et al.’s comprehensive study indicated that the most common presentations of PL-7 antibody-positive ASS were interstitial lung disease (77.8%), myositis (75%), and arthritis (56%) (19). Additional research suggests that patients with anti-PL-7 or anti-PL-12 antibodies typically experience more severe ILD, while myositis is less frequent (20). A meta-analysis of 27 studies on ASS found that joint pain and ILD were predominant, with myositis being less common among patients with anti-PL-7 or anti-PL-12 antibodies compared to those with anti-Jo-1 antibodies (21).

The patient initially presented with fever, followed by muscle pain and Gottron’s rash. Laboratory tests revealed significantly elevated serum creatine kinase levels, while chest CT indicated interstitial lung changes. Electromyography showed myogenic abnormalities, and the myositis antibody panel identified positive anti-PL-7 antibodies. Together with findings from muscle MRI and biopsy, these results confirmed a diagnosis of anti-synthetase syndrome. Given the scarcity of reported PL-7 positive ASS cases in children, differences in clinical manifestations, signs, or laboratory tests between children and adults remain unclear. This patient is the youngest reported case with PL-7 positive ASS to date. The patient’s characteristics align with those reported in existing literature, although fever as an initial symptom is atypical for previously reported cases. Earlier studies suggest that patients with non-Jo-1 positive ASS often exhibit earlier onset and more severe ILD, leading to poor prognosis and reduced survival rates. This patient did not display typical clinical manifestations of ILD, such as cough, chest pain, fatigue, or breathing difficulties, and no significant positive signs were observed in the lungs. However, interstitial changes were noted on chest CT. Consequently, ongoing treatment and follow-up are necessary to monitor lung lesions dynamically and enhance long-term prognosis. These observations may be attributed to the brief duration of the disease and follow-up period. Whether there are differences between PL-7 positive ASS cases in children and adults requires additional observation, and further case summaries are needed. Importantly, JDM, the most common form of IIM in children, is often seronegative, which complicates its diagnosis compared to adult-onset myositis. This case reinforces the need for clinicians to rely on comprehensive clinical assessment rather than antibody positivity alone in pediatric IIM diagnosis. While the EULAR/ACR criteria have improved diagnostic specificity for IIMs, their applicability in children, particularly in seronegative cases, remains an area requiring further study (7).

Currently, the preferred treatment for antisynthetase syndrome is glucocorticosteroids (21, 22), with monthly dosage reductions to achieve the minimal effective dose for improving muscle function. Hormone therapy should continue for at least 2 years. For patients presenting with severe symptoms, high-dose corticosteroids remain the first-line treatment to mitigate inflammation. Concurrent treatment with methotrexate and mycophenolate mofetil may be adjusted based on the patient’s condition. Rituximab and other biologic agents may be considered for refractory cases. The child has been treated with steroids and methotrexate, resulting in well-controlled symptoms. And recent tests show that creatine kinase levels have returned to normal. The dosage of corticosteroids is being gradually tapered.

## Conclusions

In conclusion, the incidence of ASS in children is extremely low, and the anti PL-7 antibody is a rare subtype of antisynthase antibodies. This case involves the youngest reported patient with anti-PL-7 positive ASS. It should be noted that patients with anti-PL-7 antibodies typically experience a chronic course and may develop severe complications associated with ILD, leading to a poor prognosis. Early detection and intervention are crucial for managing symptoms and enhancing outcomes. Thus, in clinical practice, patients presenting with relevant symptoms should undergo prompt laboratory testing, particularly myositis antibody profiling, to facilitate early diagnosis and treatment, thereby improving their long-term prognosis.

## Data Availability

The original contributions presented in the study are included in the article/supplementary material. Further inquiries can be directed to the corresponding author.
